# Multi-Omics Analysis of the Microbiome and Metabolome Reveals the Relationship Between the Gut Microbiota and Wooden Breast Myopathy in Broilers

**DOI:** 10.3389/fvets.2022.922516

**Published:** 2022-06-23

**Authors:** Kelang Kang, Nanxuan Zhou, Weishi Peng, Fang Peng, Mengmeng Ma, Liwei Li, Fuyi Fu, Shuhan Xiang, Haihan Zhang, Xi He, Zehe Song

**Affiliations:** ^1^College of Animal Science and Technology, Hunan Agricultural University, Changsha, China; ^2^Ministry of Education Engineering Research Center of Feed Safety and Efficient Use, Changsha, China; ^3^Hunan Engineering Research Center of Poultry Production Safety, Changsha, China; ^4^Hunan Co-Innovation Center of Animal Production Safety, Changsha, China

**Keywords:** wooden breast myopathy, meat quality, gut microbiome, metabolomics, plasma metabolomics, broilers

## Abstract

Wooden breast (WB) is a widely prevalent myopathy in broiler chickens. However, the role of the gut microbiota in this myopathy remains largely unknown, in particular the regulatory effect of gut microbiota in the modulation of muscle metabolism. Totally, 300 1-day-old Arbor Acres broilers were raised until 49 days and euthanized, and the breast filets were classified as normal (NORM), mild (MILD), or severe wooden breast (SEV). Birds with WB comprised 27.02% of the individuals. Severe WB filets had a greater L^*^ value, a^*^ value, and dripping loss but a lower pH (*P* < 0.05). WB filets had abundant myofiber fragmentation, with a lower average myofiber caliber and more fibers with a diameter of <20 μm (*P* < 0.05). The diversity of the intestinal microflora was decreased in birds with severe WB, with decreases in *Chao 1*, and observed species indices. At the phylum level, birds with severe WB had a lower Firmicutes/Bacteroidetes ratio (*P* = 0.098) and a decreased abundance of Verrucomicrobia (*P* < 0.05). At the species level, gut microbiota were positively correlated with 131 digesta metabolites in pathways of glutamine and glutamate metabolism and arginine biosynthesis but were negatively correlated with 30 metabolites in the pathway of tyrosine metabolism. In plasma, WB induced five differentially expressed metabolites (DEMs), including anserine and choline, which were related to the severity of the WB lesion. The microbial-derived metabolites, including guanidoacetic acid, antiarol, and (2E)-decenoyl-ACP, which entered into plasma were related to meat quality traits and myofiber traits. In summary, WB filets differed in gut microbiota, digesta, and plasma metabolites. Gut microbiota respond to the wooden breast myopathy by driving dynamic changes in digesta metabolites that eventually enter the plasma.

## Introduction

Wooden breast (WB) is a breast myopathy that has recently begun to occur in the pectoral muscles of broiler chickens at slaughter age. Wooden breast-affected filets are characterized by a dull appearance and tough texture (1). It has been reported that birds with wooden breast were even up to 79% (2), which implies chicken breast filets with low quality has become one of the major problems nowadays, and it has been estimated to cause annual economic losses up to one billion dollars worldwide (3). Several investigations have demonstrated potential factors involved in the pathogenesis of this myopathy (4), including excessive growth rate, oxidative stress (5), ischemia with resultant local hypoxia (6), and lipid and glucose metabolism (7, 8).

Many studies have investigated the host–microbiota interactions concerning metabolism, signaling, and immune-inflammatory axes that physiologically connect the gut, liver, muscle, and brain (9). Furthermore, studies have confirmed that gut microbial populations are important for the growth, mass, and function of skeletal muscles using germ-free mice, gnotobiotic mice, and fecal microbiota transplants as models (10–12). The diversity and stability of gut microbiota are intimately linked to mass, strength, and movement capability of muscle in mammals (13). Balanced gut microbiota are involved in the production and secretion of short-chain fatty acids (SCFAs) (14), vitamins (15), bile acids (16), and amino acids, and their metabolites are involved in a series of myopathies, such as sarcopenia (17), myasthenia gravis, and other neuromuscular dysfunctions (18). However, gut microbiota and its relationship with animal health and productivity in commercial broiler chickens have been difficult to establish due to the high variability between flocks. Many factors like environment, nutrition, and host factors influence a multitude of commensal and pathogenic microbes surrounding birds during their growth cycle in the farms. But the literature provides some evidence about WB and cecal gut microbiota in birds. Maharjan reported that unclassified *Lactobacillus* had a relatively higher abundance in a WB myopathy group, whereas *Lactobacillus acidipiscis* was identified in non-myopathic birds (19). Zhang et al. (20) reported that *Selenomonas bovis* and *Bacteroides plebeius* were the two microbes with the highest abundance in the cecum of WB birds, and the microbiota of WB birds had reduced glycolysis and urea cycles but an increased tricarboxylic acid (TCA) cycle. These studies have reported differences in the gut microbiota composition in birds with WB. Therefore, the gut microbiota may affect the progression of WB myopathy. However, relatively little is known about the contribution of the gut microbiota to WB myopathy, and the mechanism underlying the effects of gut microbiota remains to be elucidated.

In this study, we sought to demonstrate the differences in gut microbiota and their metabolites between three states—a normal state, mild WB, and severe WB—to reveal the differences in gut microbial metabolites that contribute to the plasma metabolome and affect the skeletal muscle. Therefore, we investigated the meat quality, histomorphological differences, and myofiber characteristics in WB filets and then analyzed the differences in gut microbiota. Finally, differential abundances between the cecal microbial metabolites and plasma metabolites associated with various degrees of WB severity in broilers were identified.

## Materials and Methods

### Ethical Statement

The study was conducted in accordance with the Regulations of the Experimental Animal Administration, approved by the Committee on the Ethics of Animal Experiments of Hunan Agriculture University (GBT2018).

### Animal Management and Sampling

Totally, 300 1-day-old Arbor Acres (AA) broiler chicks were raised at a chicken house at the Hunan Agricultural University Poultry Research Farm. All birds were kept to 30 cages randomly, with 10 birds per block. Water and feed were provided *ad libitum* to all birds, with 23/1-h light/dark cycle throughout the whole study. All birds were fed with corn–soybean-based feed, in two feeding phases: starter to grower diets (days 0–21) and finishing diets (days 22–49). The diet was formulated in accordance with NRC nutrition recommendations (NRC, 1994) and raised under standard protocols for AA broilers. The chickens were subjected to a routine vaccination program. All birds selected for sampling and analysis were healthy. On day 49, before killing, blood was collected in a 10-ml fresh tube with EDTA from the wing vein to get plasma. Then the birds were all euthanized and evaluated using the WB myopathy scoring system based on the area of palpable firmness, reported by Sihvo et al. (21). For short, we manually palpated and classified 300 chicken filets into three kinds of breast filets based on the texture and firmness: normal breast (NORM), mild wooden breast (MILD), and severe wooden breast (SEV). There was no toughness or hardness area in normal pectoralis major filets (NORM); the mild wooden breast pectoralis major filets (MILD) had toughness <50% of total pectoralis major filets, in the cranial aspect mainly; the severe wooden breast pectoralis major filets (SEV) had toughness more than 50% of total pectoralis major filets in both cranial and caudal aspects of filets and exhibited diffuse pallor and multifocal and visible sclerotized protrusion. The cecal contents were collected, immediately frozen in liquid nitrogen, and stored at −80°C for 16S rRNA sequencing analysis and metabolomics subsequently. For 16S sequencing and metabolomics, 20 samples each for NORM, MILD, and SEV groups.

### Meat Quality Analysis

The meat color (L, a^*^, and b^*^ values) was measured by a portable chromameter (CR 400, Minolta, Osaka, Japan) from three random readings at 45 min postmortem, following the manufacturer's manual. The average pH value of each breast filet meat sample was measured *via* a digital pH meter (Testo 205, Testo AG, Germany) at 45 min postmortem, in which three points were randomly measured, and an average value was obtained. The shear force value (N) was calculated as the maximum force recorded during shearing, by Texture Analyzer TA.TXplus (Stable Micro Systems, Surrey, England). Drip loss was expressed as a percentage of the weight loss over initial meat sample weight after storage at 4°C for 24 h. Press loss was measured as described by Prieto et al. (22).

### Histology

All pectoralis muscle (breast muscle) samples for histology were obtained as described by Wang et al. (23). In brief, breast muscle filets were dissected perpendicular to the muscle fiber direction and tied tightly to wooden applicator sticks to avoid contraction. The samples were fixed in 10% neutral formalin and stored. After 24 h of formalin immersion, the samples were dehydrated through an alcohol gradient and embedded in a paraffin block. Subsequently, the paraffin blocks of filets were sectioned at 5 μm and stained with hematoxylin and eosin (H&E). A light microscope fitted with a digital camera was used to take photographs of the myofibers in 100×.

The myofiber diameter and myofiber cross-sectional area were measured from three photomicrographs as previously described (23, 24). At least 40 measurements were taken in each micrograph using ImageJ software (ImageJ Fiji, https://imagej.net/Fiji). Two categories of myofiber calibers were counted: fiber width <20 μm and fiber width >70 μm.

### Estimation of Antioxidant Enzyme Activities and Oxidative Damage Biomarkers

The total antioxidant capacity (T-AOC), total superoxide dismutase (T-SOD), malondialdehyde (MDA), catalase (CAT), and glutathione peroxidase (GSH-Px) were assessed by using kits from Nanjing Jiancheng Bioengineering Institute, Jiangsu, China. All procedures were conducted according to the manufacturers' instructions. The concentration of 8-hydroxy-2′-deoxyguanosine (8-OHdG) and glutathione (GSH) were determined with an ELISA kit (Ameko, Shanghai, China).

### Microbial Community DNA Isolated and 16S rRNA Sequencing Analysis

In chickens, cecal microbiota is responsible for the plethora of microbes and metabolites in the chicken intestine, which could protect the gut microbiota to an extent. The total microbial DNA was extracted from cecal digesta by using the CTAB method (25). Later, the concentration, quality, and integrity of DNA were determined. Then the total DNA was diluted to 1 ng/μl using sterile water prior to PCR amplification. To amplify the microbial 16S rRNA genes, PCR was performed using DR PCR mastermix (Pyrobest DNA Polymerase. TaKaRa. DR500A) with a pair of universal forward and reverse primer sets for 16S V3-V4 regions (26). The forward sequence was 5′-TCGTCGGCAGCGTCAGATGTGTATAAGAGACAGCCTACGGGNGGCWGCAG-3′; the reverse primer sequence was 5′-TCTCGTGGGCTCGGAGATGTGTATAAGAGACAGGACTACHVGGGTATCTAATCC-3′. After purification of PCR amplicons, libraries for microbial 16S rRNA gene sequences were constructed, and sequencing was performed using the Illumina NovaSeq6000 platform. Raw sequence data were filtered and denoised, and low-quality regions of sequences were trimmed and removed. The QIIME2 (v2019.4) platform was used to generate the amplicon sequence variant (ASV) feature table, and rarefaction curves and alpha diversity and beta diversity analyses was carried out. For taxonomic classification, the Greengenes database (version 13.8, http://greengenes.secondgenome.com/) was selected and aligned (27, 28). Principle coordinate analysis (PCoA) was performed using the Bray–Curtis dissimilarity metric.

### LC-MS Untargeted Metabolomics

The metabolome of cecal digesta or plasma (*n* = 20 for each group) from birds with different severities of WB was analyzed *via* the untargeted *LC-MS*-based metabolomics approach. Cecal digesta were homogenized with methanol and centrifuged at 4°C, 12,000 rpm for 10 min. Then the supernatant was transferred, dried in vacuum, and dissolved with 200 μl 2-chlorobenzyl acetonitrile solution, and the supernatant was filtered through a 0.22-μm membrane to obtain the prepared samples for LC-MS. Quality control (QC) samples were prepared by mixing 20 μl aliquots from each sample to monitor deviations of the analytical results from these pool mixtures.

Metabolic profiling was performed using LC-MS. Chromatographic separation was accomplished in a Thermo Vanquish system equipped with an ACQUITY UPLCR® HSST3 column. The ESI-MSn experiments were carried out on the Thermo Exactive mass spectrometer. Dynamic exclusion was implemented to remove unnecessary information in MS/MS spectra.

Raw data files were converted to an mzXML format by ProteoWizard (v3.0.8789) (29), and the XCMS package in R (v3.3.2) was used to identify the peaks, peak filtration, and peak alignment with the following parameter: bw = 2, ppm = 15, peakwidth = c (5, 30), mzwid = 0.015, mzdiff = 0.01, and method=centWave. After that, the matrix of the mass-to-charge ratio (*m*/*z*), retention time (rt), and intensity was exported, and data from positive and negative ion modes were analyzed separately. The metabolites were confirmed on the basis of their exact molecular weights, and the possible empirical formulae of the metabolites were speculated (molecular weight error <30 ppm). Then metabolites were annotated by *m*/*z* and MS/MS fragmentation matches, and one compound was annotated by *m*/*z* and RT match. All statistical analyses downstream were performed using MetaboAnalyst 4.0 web tool (https://www.metaboanalyst.ca/MetaboAnalyst/faces/home.xhtml), as described by Gururaj (30). Metabolic pathway enrichment analysis of these identified metabolic biomarkers was carried out by MetaboAnalyst 4.0, based on KEGG, with the MetPA function, and tested through a hypergeometric test for determining significance. The same methodology was followed for plasma samples. To investigate the metabolite that could be influenced by gut microbe, the Pearson correlation between the abundance of gut microbe and digesta metabolites was calculated. Pearson correlations were also determined between studied parameters of microbial metabolites and meat quality traits, like myofiber characteristics.

### Statistical Analysis

Statistical analyses were carried out by Prism V9.0.0 (https://www.graphpad.com/scientific-software/prism/). Comparisons between groups were performed using one-way analysis of variance (one-way ANOVA). Differences between two groups were tested by independent samples *t*-tests. Data are expressed as mean ± SE. A *P*-value < 0.05 was considered statistically significant. The graph was plotted using Prism.

## Results

### Appearance, Meat Quality, and Antioxidant Capacity of Wooden Breast

**Figure 1:**
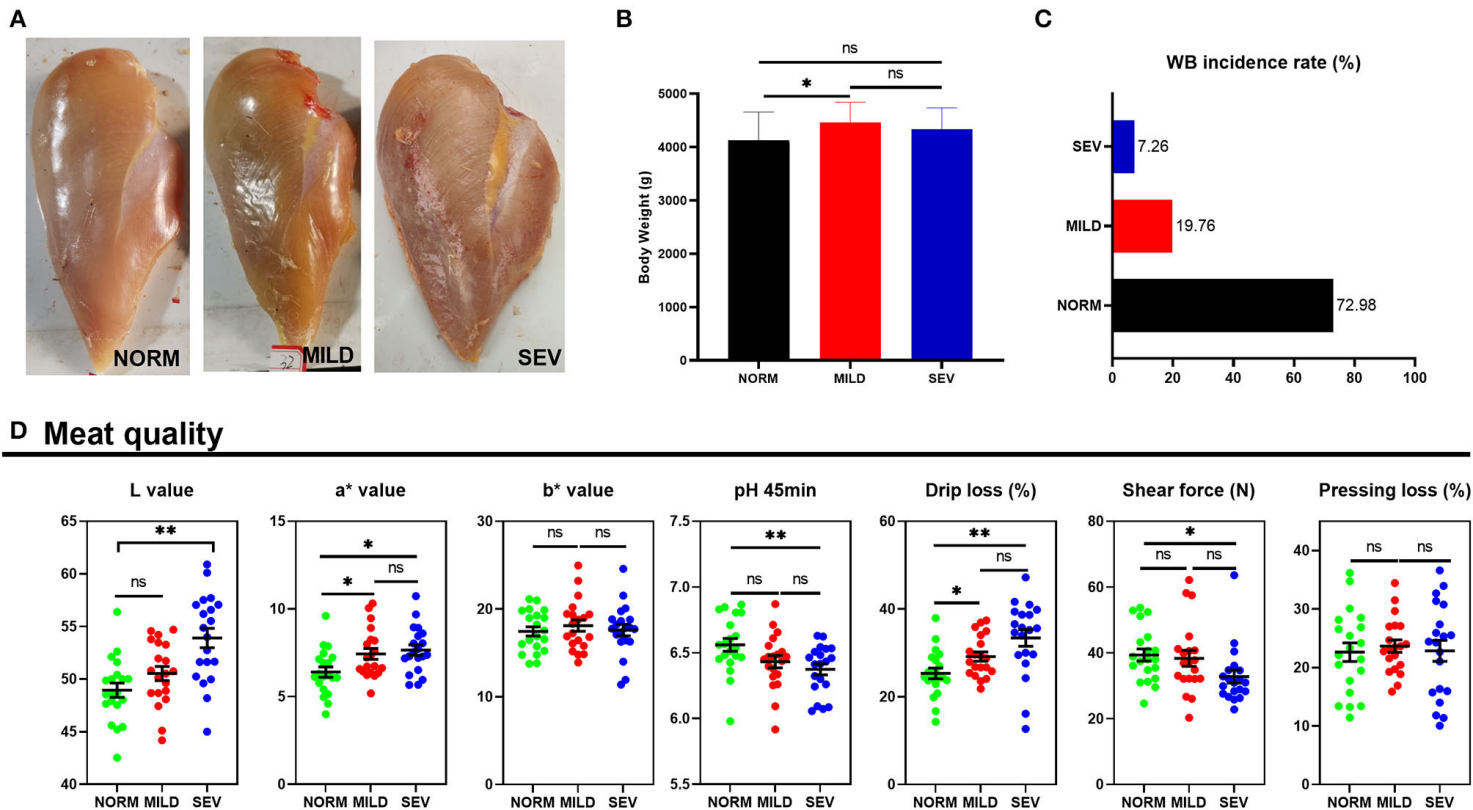
Appearance, incidence, and meat quality of WB. **(A)** Appearance of normal, mild, and severe WB filets. **(B)** Body weight of birds with or without WB. **(C)** Incidence of WB. **(D)** Meat quality of WB filets, L value, lightness, a* value, redness, b* value, and yellowness. “*” means *P*-value < 0.05; “**” means *P*-value < 0.01.

The representative appearance of WB filets is shown in Figure 1A. The severity of WB was less profoundly affected by body weight, among which birds with mild WB had higher body weight than normal birds (Figure 1B). In terms of the incidence of WB, birds with WB comprised 27.02% (a total of 248 birds, mild WB, 19.76%, and severe WB, 7.26%; Figure 1C). The severe WB filets had higher L^*^ values (light values) than both mild and normal filets (*P* < 0.01, Figure 1D). Both severe and mild WB filets had higher a^*^ values (redness values) than normal filets (*P* < 0.05), but no significant difference was observed between mild and severe WB filets (*P* > 0.05). The pH (45 min) of severe WB filets was significantly decreased compared to normal filets (*P* < 0.01). Both severe and mild WB filets had higher drip loss than normal filets (*P* < 0.05), but there was no difference between the two groups (*P* > 0.05). The severe WB filets had lower shear force than normal filets (*P* < 0.05). There was no significant difference in the b^*^ value (yellowness values) or pressing loss among the three groups (*P* > 0.05). The severe WB filets exhibited an increased content of GSH and MDA and higher T-SOD and CAT (Table 1, *P* < 0.05).

**Table 1:** Effect of wooden breast severity on antioxidant traits in pectoralis major filets^1^.

**Traits**	**NORM**	**MILD**	**SEV**	**SEM**	***P*-value**
**Muscle**					
T-AOC, U/mgprot	0.511	0.477	0.668	0.034	0.120
T-SOD, U/mgprot	140.0^b^	178.5^b^	290.2^a^	16.7	<0.001
CAT, U/mgprot	12.5^b^	18.0^ab^	21.9^a^	1.44	0.006
GSH-PX, μM/mgprot	130.7	133.4	177.7	9.61	0.092
GSH, μg/mgprot	40.8^b^	50.0^b^	67.5^a^	3.06	0.001
8-OHdG, ng/mgprot	10.19	11.66	10.54	0.31	0.130
MDA, nM/mgprot	2.06^c^	2.63^b^	3.33^a^	0.11	<0.001
**Serum**					
T-AOC, U/ml	7.61^a^	5.91^ab^	4.28^b^	0.452	0.013
T-SOD, U/ml	129.9^b^	148.4^ab^	160.3^a^	4.8	0.029
CAT, U/ml	12.87	12.06	12.19	0.32	0.548
GSH-PX, μM/L	707.8^a^	693.7^a^	515.8^b^	27.5	0.007
GSH, μg/ml	146.4	142.2	142.3	1.82	0.591
8-OHdG, ng/ml	16.41	18.95	21.93	1.13	0.138
MDA, nM/L	3.25	3.52	2.54	0.21	0.159

a−b *Values within a row without a common letter are significantly different (P < 0.05), Duncan test*.

1 *NORM, normal filets; MILD, mild wooden breast filets; SEV, severe wooden breast filets, n = 60*.

### Histopathologic Studies and Myofiber Parameters in Wooden Breast Myopathic Birds

**Figure 2:**
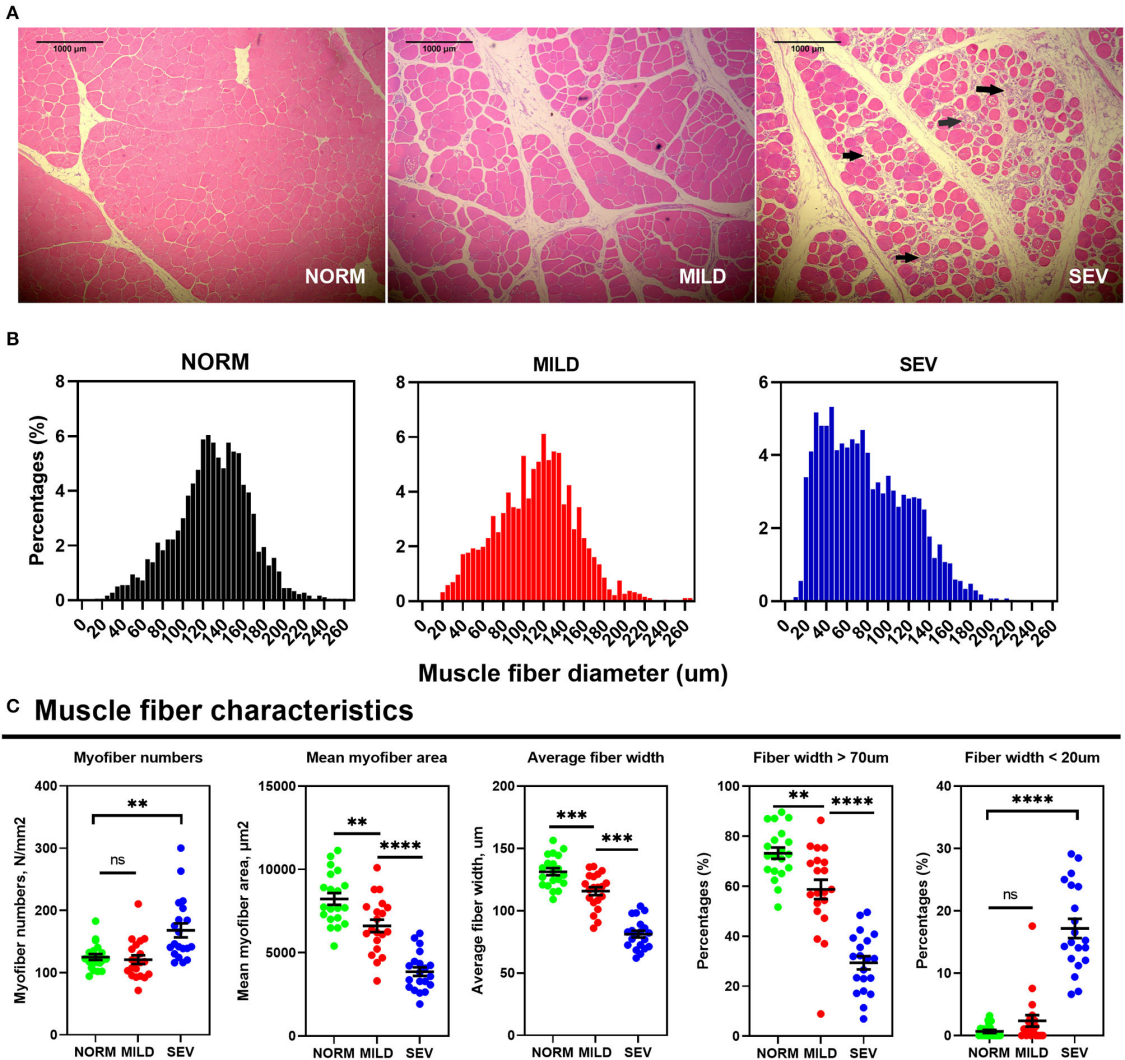
Histopathologic studies and myofiber parameters in WB filets. **(A)** Breast filet muscle tissue sections were stained with H&E and examined under a microscope. Left, normal filets; middle, mild WB; right, severe WB. **(B)** Histogram of muscle fiber diameters. **(C)** Muscle fiber characteristic. “***” means *P*-value < 0.001; “****” means *P*-value < 0.0001.

Figure 2A presents representative photomicrographs showing the morphological structure of WB filets. The fiber atrophy and infiltration of inflammatory cells are indicated by the arrowhead. Birds with WB showed a discernible decrease in the myofiber caliber compared with normal birds (Figures 2B,C). In detail, the myofiber numbers per unit in SEV filets were greater than those in the NORM and MILD filets, with abundant myofiber fragmentation (*P* < 0.01). By contrast, the mean myofiber area and average myofiber width in SEV filets were lower than those in the NORM and MILD filets (*P* < 0.001). The percentage of fibers with a diameter <20 μm increased significantly with increasing WB lesion severity (*P* < 0.001), while the percentage of fibers with a diameter >70 μm decreased significantly in both mild and severe WB filets (*P* < 0.01). In severe WB filets, only 29.34% of the myofibers had a diameter >70 μm. In NORM filets, this ratio reached as high as 79.13%, a value that was much higher than that in MILD and SEV WB filets.

### Composition and Diversity of Cecal Microbiota in Wooden Breast Birds

**Figure 3:**
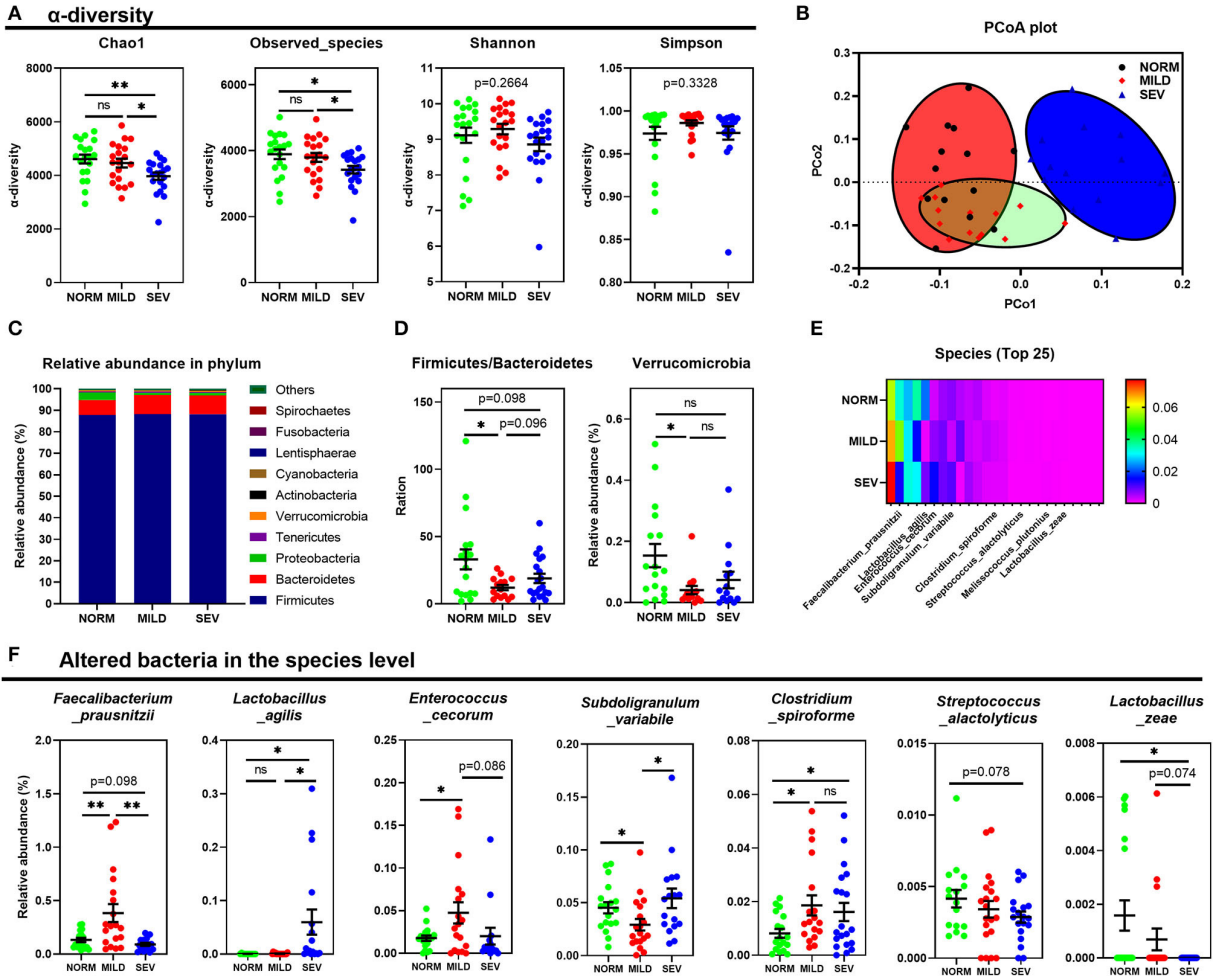
Composition and diversity of cecal microbiota in birds with WB. **(A)** Alpha diversity index analysis. **(B)** PCoA of composition of gut microbiota between birds with or without WB. **(C)** Composition of gut microbiota at the phylum level. **(D)** Difference in the relative abundance of microbes between birds with or without WB in the phylum level. **(E)** Heat map of microbiota on the abundance of top 25 between birds with or without WB in the species level. **(F)** Details of WB altered bacteria in the species level. “*” means *P*-value < 0.05; “**” means *P*-value < 0.01.

Alpha diversity analysis indicated that birds with severe WB had lower microbial community richness and diversity than normal birds without WB myopathy, with a lower *Chao 1* index and fewer observed microbial species than normal birds and birds with mild WB (*P* < 0.05, Figure 3A). However, comparison between birds with and without WB lesions demonstrated no significant differences in Shannon or Simpson indices (*P* > 0.05). The beta diversity of gut microbiota showed clear differences between birds in NORM, MILD, and SEV groups (Figure 3B). Areas of NORM colocalized with areas of MILD, as shown under red and green staining, but SEV was isolated (blue, Figure 3B).

Microbial profiles of samples were analyzed at the phylum level, and the Firmicutes/Bacteroidetes ratio was decreased significantly in birds with mild WB (*P* < 0.05, Figures 3C,D, left). There was also a downward trend in the Firmicutes/Bacteroidetes ratio in birds with severe WB compared to normal birds (*P* = 0.098). In addition, the abundance of *Verrucomicrobia* also decreased in birds with mild WB (*P* < 0.05, Figure 3D, right). The WB severity-dependent composition of the gut microbiota at the species level is shown in Figure 3E. For example, the MILD group had a significantly higher abundance of *Faecalibacterium prausnitzii* than the other two groups (*P* < 0.01); the relative abundance of *Lactobacillus agilis* in birds with severe WB was higher than that in the NORM and MILD groups (*P* < 0.05); *Enterococcus cecorum* was enriched in the MILD group, while the abundance of *Subdoligranulum variabile* in the MILD group was lower than that in NORM and SEV (*P* < 0.05). The abundance of *Clostridium spiroforme* in both MILD and SEV groups was higher than that in NORM (*P* < 0.05); the abundance of *Streptococcus alactolyticus* in the SEV group showed a decreased trend compared to the NORM group (*P* = 0.078), and the abundance of *Lactobacillus zeae* decreased significantly with the severity of WB lesions (Figure 3F).

### Cecal Microbiota-Related Cecal Metabolites

**Figure 4:**
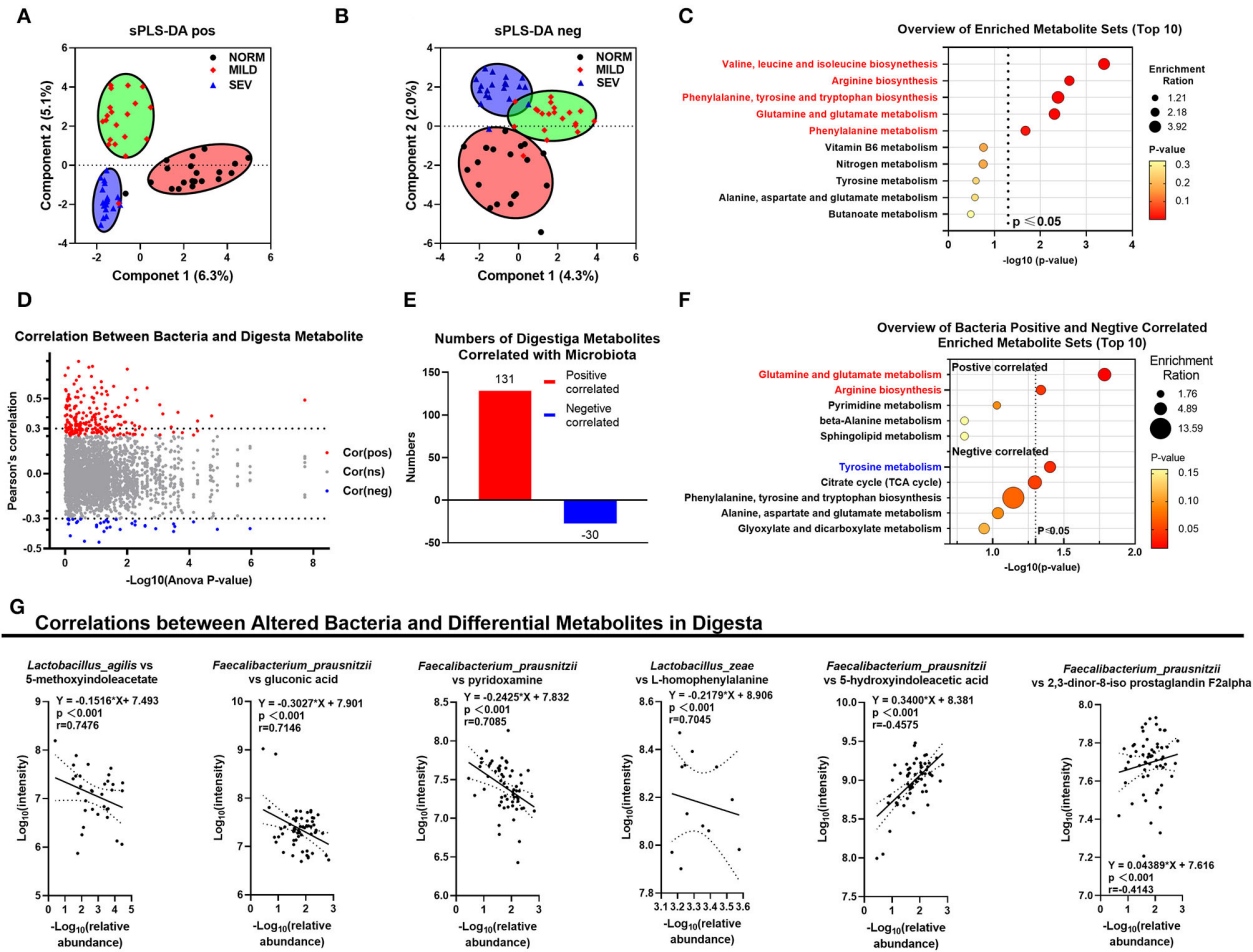
Results of digesta metabolomics. **(A)** sPLS-DA analysis of positive ions. **(B)** sPLS-DA analysis of negative ions. **(C)** GO pathway annotation in digesta metabolites. **(D)** Correlation between bacteria and digesta metabolites; red points, positively correlated; blue, negatively correlated; gray, not significantly correlated. **(E)** Numbers of digesta metabolites correlated with microbiota. **(F)** GO pathway annotation for bacteria-related metabolites. **(G)** Correlation between altered bacteria and differential metabolites in digesta.

The metabolites in the gut microbiota exhibited a stepwise progression, and segregation occurred from normal to severe WB (sPLS-DA analysis, Figures 4A,B). Those digesta metabolites were enriched significantly in the GO pathways of valine, leucine, and isoleucine biosynthesis; arginine biosynthesis; phenylalanine, tyrosine, and tryptophan biosynthesis; glutamine and glutamate metabolism; and phenylalanine metabolism (*P* < 0.05, Figure 4C).

First, we assume that digesta metabolites correlated with cecal microbiota. The correlation between the abundance of bacteria in species of the top 25 digesta metabolites was visualized using volcano plots (Figure 4D). A total of 131 metabolites were positively correlated with the gut microbiota mentioned earlier, and 30 metabolites were negatively correlated with the same groups (Figure 4E, Supplementary Table S3). In the GO functional annotation, the gut microbiota were positively correlated with the pathways of glutamine and glutamate metabolism (with metabolites of l-glutamic acid) and arginine biosynthesis but negatively correlated with the pathway of tyrosine metabolism (Figure 4F). Correlations between microbial populations and metabolites were identified (Figure 4G); these included *L. agilis* and 5-methoxyindoleacetate (*r* = 0.7476, *P* < 0.001), *F. prausnitzii* and gluconic acid (*r* = 0.7146, *P* < 0.001), *F. prausnitzii* and pyridoxamine (*r* = 0.7085, *P* < 0.001), *L. zeae* and l-homophenylalanine (*r* = 0.7045, *P* < 0.001), *F. prausnitzii* and 5-hydroxyindoleacetic acid (*r* = −0.4575, *P* < 0.001), and *F. prausnitzii* and 2,3-dinor-8-iso prostaglandin F2α (*r* = −0.4143, *P* < 0.001).

### Plasma Metabolite Alternations by Cecal Metabolites in Wooden Breast Birds

**Figure 5:**
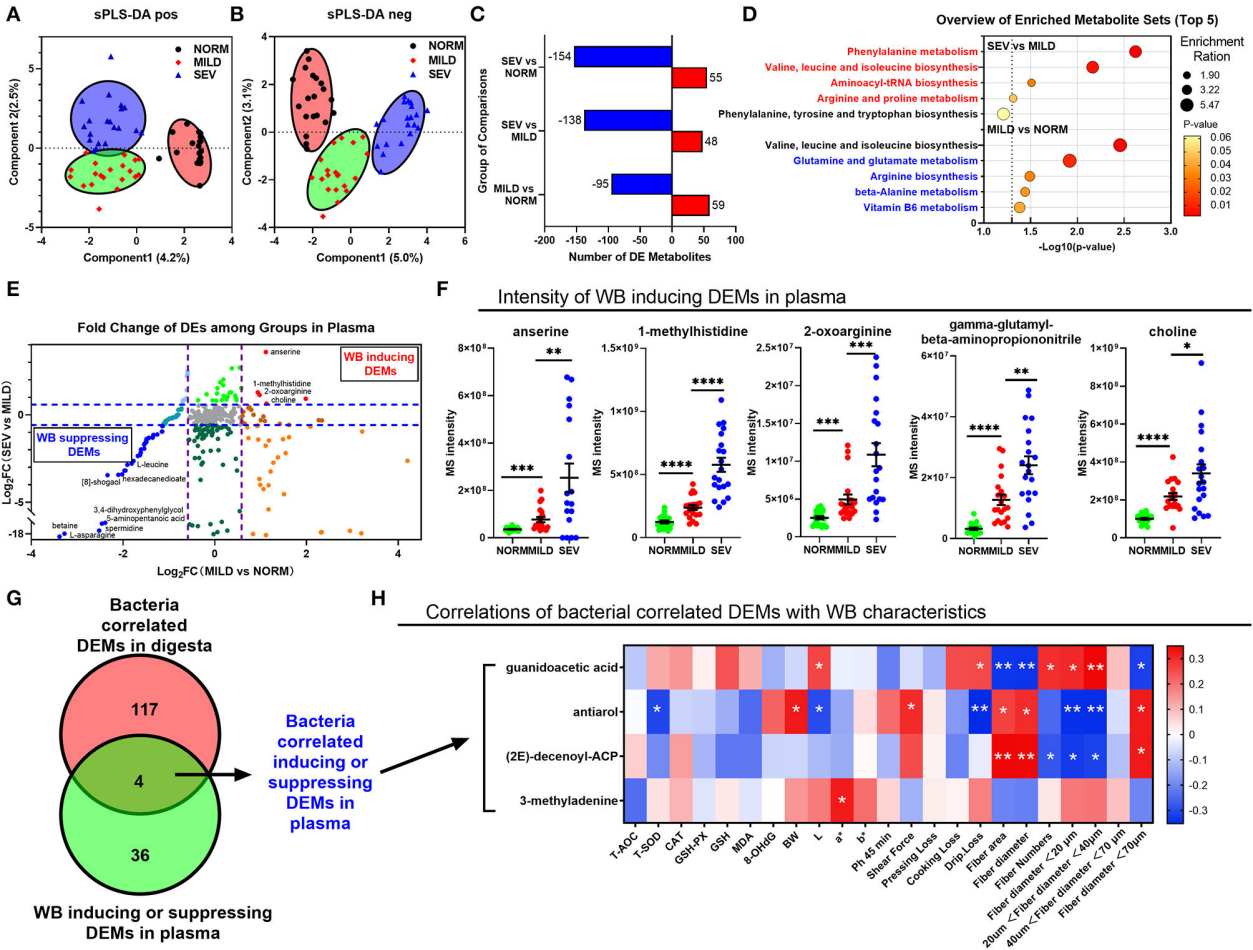
Results of plasma metabolomics and correlation analysis with digesta metabolomics. **(A)** sPLS-DA analysis of positive ions. **(B)** sPLS-DA analysis of negative ions. **(C)** Bar graphs for the number of differential expressed metabolites. **(D)** GO pathway annotation for differential expressed metabolites between normal and mild WB; between severe and mild WB. **(E)** Coordinate plot for log_2_ (fold change value) for metabolites between mild WB and normal in *X*-axis; for metabolites between severe and mild WB in *Y*-axis. **(F)** Details of intensity of WB-inducing DEMs in plasma. **(G)** Venn diagram of metabolites between bacteria correlated DEMs in digesta and WB-inducing or -suppressing DEMs in plasma. **(H)** Correlation of bacteria correlated DEMs with WB characteristics of antioxidant properties, meat quality, and myofiber characteristics. *P < 0.05; **P < 0.01;***P < 0.001. ns, not significant.

There was a distinct composition of both gut microbial-related cecal metabolites and serum metabolites in normal birds compared with the severe WB birds, with mild WB birds being intermediate between the two (Figures 5A,B), coincident with the results of digesta metabolites. The numbers of differential metabolites between normal birds and mild and severe WB are presented in Figure 5C. In birds with severe and mild WB, phenylalanine metabolism; valine, leucine, and isoleucine biosynthesis; aminoacyl-tRNA biosynthesis; and arginine and proline metabolism were altered. In comparison between normal birds and birds with mild WB, the pathways involved valine, leucine, and isoleucine biosynthesis; glutamine and glutamate metabolism; arginine biosynthesis; beta-alanine metabolism; and vitamin B6 metabolism (Figure 5D). Metabolites in the plasma demonstrated upregulation in birds with normal to mild to severe WB and, thus, could be defined as being differentially expressed metabolites (DEMs) correlated with the severity of the WB lesion, including anserine, 1-methylhistidine, 2-oxoarginine, γ-glutamyl β-aminopropiononitrile, and choline (Figures 5E,F). Conversely, the WB-suppressing DEMs included l-leucine, hexadecanedioate, 8-shogaol, 3,4-dihydroxyphenylglycol, and l-asparagine; four metabolites were correlated both with gut microbiota in the digesta (Figure 4E) and WB inducing or suppressing in plasma (Figure 5E). These could be identified as gut microbiota correlated with inducing or suppressing DEMs in plasma (Figure 5G). The microbial-related metabolites guanidoacetic acid, antiarol, and (2E)-decenoyl-ACP were significantly related to meat quality traits and myofiber traits (Figure 5H). The metabolite 3-methyladenine was only significantly positively correlated with L traits. Only antiarol was positively correlated with the enzyme of total superoxide dismutase (T-SOD) in breast muscles.

## Discussion

Wooden breast filets lead to decreased consumer preference, thereby posing a significant challenge to breeders and causing economic losses in the broiler industry. Normally, selection for the accelerated growth rate and high breast yield in broiler chickens has been regarded as association with an increase in myopathies (31). In the present study, birds with mild WB had a higher body weight gain than normal birds with the same feeding conditions. But the body weight did not increase with the severity of the lesion of WB, which indicates the severity of WB may not markedly affected by body weight. The meat quality of WB as measured was consistent with that of a previous study (32, 33). A higher L value, a^*^ value, and drip loss, as well as lower pH postmortem, were observed in the WB filets. A high lightness value is considered to be an indicator of the paleness of breast meat (34) and also indicates a higher amount of exudate, causing light scattering from the meat surface (35). Identification of color is an easy way to determine the pH of meat: very dark meat will have a high pH, whereas light meat will have a low pH (36). It has been reported that the pH and color of pectoralis major muscles of broilers with severe WB are similar to pale, soft, exudative (PSE) meat (37) and are characterized by low pH, a pale and exudative appearance, and a soft texture (38). Normally, when the concentrations of glucose and lactate in the meat are lower, there is a lower postmortem pH than normal meat (39). This explains the decreased tendency of pH postmortem in severe WB meat compared to normal meat. The WB filets also presented a poor water-holding capacity, with higher drip loss. Normally, the juiciness of the chicken breast, including tenderness and water-holding capacity, is among the most important attributes of meat quality as they substantially affect consumer satisfaction (40). In conclusion, our results indicate that severe wooden breast results in poor meat quality, affecting the consumers' assessment. Meat quality has a close relationship with muscle antioxidant status. One of oxidative stress parameters, MDA, increased in WB meat, which indicates a lipid metabolism perturbation (41), but the enzymes of antioxidant still work to remove oxygen free radicals, reduce lipid peroxidation, and protect myocytes.

Recently, morphological changes have been demonstrated in wooden breast filets (33, 42). Previous results concerning such changes were similar to our observations in the present study; these changes included visible cytoplasmic vacuolation, atrophy, degenerative sarcolemma, and infiltration of inflammatory cells. Generally, the total number of myofibers remains unchanged after birth (43). Rather than adding myofibers, muscles grow longitudinally through the accretion of myoblasts, resulting in an increasing pool of myonuclei, which regulate the addition of sarcomeric units and skeletal muscle growth (44, 45). Intense selection for growth performance in broilers causes an excessive rate of breast muscle growth (46), which may favor more vulnerable myofibers. After the occurrence of myofiber injury, monocytes invade and remove necrotic cells and cellular debris (47). These results were consistent with those of H&E staining in the present study as abundant mononuclear cell infiltrates were observed. In the normal physiological status, the fragmentation of myofibers can result from full myofiber replacement or local damage and repair (48), but in severe WB, the fragmented myofibers occupied the vast majority, indicating that loss of repair capacity may contribute to these events. In addition, the subtle balance of removing and repairing in muscles was perturbed; this could explain the accumulation of damaged myofibers. Skeletal muscle fibers as factors for meat quality (49). Hence, fragmented myofibers in severe WB was responsible for a bad water-holding capacity and other alternation in meat quality.

Gut microbiota play a fundamental role in maintaining normal intestinal function and regulating host metabolism (50). Previous work has shown that bacterial communities can be associated with this musculoskeletal disorders. Maharjan reported that WB results in apparent changes in the microbial flora (19). Zhang et al. (20) reported that *S. bovis* and *B. plebeius* were the two microbes with the highest abundance in the cecum of WB birds, and the microbiota of WB birds had reduced glycolysis and urea cycles but increased tricarboxylic acid (TCA) cycle. Here, we demonstrated a variety of contrasts between birds with mild WB and severe WB and evaluated those distinctions. The *Chao 1* biodiversity indices and observed species reflect the α-diversity of gut microbiota, which both decreased in birds with WB. The Firmicutes/Bacteroidetes ratio was decreased in birds with WB, a pattern which is often regarded as a marker of obesity (51), and an increased or decreased Firmicutes/Bacteroidetes ratio is an important feature of dysbiosis (52). In the colitis model, it was also an important reflection of balance between gut microbiota and intestinal mucosal (53). At the species level, *C. spiroforme*, an opportunistic pathogen found in rabbits, which produces a binary toxin and similar to the iota toxin (54), increased significantly in birds with WB, while the *L. zeae*, a probiotic reported by Lambo (55), was decreased in birds with severe WB, as well as *S. alactolyticus*. The *S. alactolyticus* was also reported to be present in high relative abundance in healthy birds but decreased in infected birds with lesions, in line with a previously report (56). Taken together, the gut microbiota of birds with WB myopathy were presented, and it may be involved in the development of WB.

The gut microbiome metabolites drive dynamic changes in digesta and enter the circulation (57). By sPLS-DA analysis, a similar chronic progressive course was observed in both digesta metabolomics and plasma metabolomics from normal breast to mild WB to severe WB. Therefore, we hypothesized that WB was interrelated with the gut microbiome composition and plasma metabolites. In other words, microbial metabolites affected the apparent concentrations of those metabolites in plasma through cecal metabolites. Valine, leucine, and isoleucine are branched chain amino acids (BCAAs), and skeletal muscles, as the initial site of BCAA catabolism, is accompanied by the release of alanine and glutamine into the bloodstream (58). *In vitro*, arginine plays a vital role in skeletal muscle fiber-type transformation from fast twitch to slow twitch *via* the Sirt1/AMPK pathway (59). Arginine was also reported to stimulate muscle protein synthesis by inducing the phosphorylation of mTOR in skeletal muscles (60). It could be speculated that microbial-derived arginine replenishes endogenous arginine synthesis. In addition, phenylalanine, tyrosine, and tryptophan biosynthesis in the myosin regulatory light chain controls non-muscle myosin II assembly and function (61); the glutamate metabolism pathway, which is essential to maintain skeletal muscle metabolism, was significantly altered with an altered metabolite, l-glutamic acid. Taken together, the results of GO annotation indicated that microbial-related digesta metabolites participated in diverse muscle activities. The Pearson correlation analysis indicated associations between microbes and metabolites, leading to a deeper understanding of how gut microbes modulate the components of digesta metabolites. The results were in line with the expectation that there is a substantial amount of association between digesta and gut microbes. Gut microbiota were positively correlated with glutamine and the glutamate metabolism and arginine biosynthesis pathways, mainly involving l-glutamic acid and pyrrolidonecarboxylic acid. The glutamine association with thyroid hormones regulates muscle weight and fiber diameter in resting and atrophic conditions and results in protection from muscle loss during atrophy (62). Furthermore, the gut microbiota is negatively related to tyrosine metabolism, where phosphorylation of tyrosine is regulated by AMPK and controls metabolism in human skeletal muscles (63). Many gut microbes coupled with digesta metabolites showed strong and significant correlations. Metabolite 5-hydroxyindoleacetic acid is a product of the kynurenine pathway of tryptophan metabolism (64), and it can be used as a biomarker for depression, hepatomegaly, bronchospasm, and cardiac disease (65). *Lactobacillus agilis* was negatively correlated with 5-methoxyindoleacetate. These results indicate that the gut microbiota participated in indoleacetic acid metabolism, which guided cross-talk between the methoxyindole and kynurenine pathways of tryptophan metabolism. Gluconic acid can reach the large intestine to stimulate lactic acid bacteria (66); this was negatively correlated with *F. prausnitzii*. In conclusion, the gut microbiota modulated changes in glutamine, arginine, and tyrosine metabolism. In addition, the finding that gut microbes were negatively related to the numbers of metabolites indicated that lack of some critical microbial-related metabolites may eventually contribute to severe WB.

In plasma, the number of DEMs presented a gradient from normal to mild to severe WB, implying a progressive deterioration in birds. Among the DEMs, the valine, leucine, and isoleucine biosynthesis pathway was associated with the WB disease progression. BCAA supplementation is often regarded as an efficient nutritional strategy to alleviate skeletal muscle damage (67), and upregulated anabolism of BCAAs also increases the mitochondrial content in cells of skeletal muscles and adipocytes (68). Phenylalanine metabolism was upregulated in severe WB compared to mild WB, similar to a report claiming that myotrophic lateral sclerosis and Duchenne muscular dystrophy (DMD) *in vivo* displayed similar symptoms (69), in which these protein changes may represent the relative loss of the long α-helical structures within muscle proteins (70). These results confirmed that the muscle damage in severe WB was still in the process of the myofiber remodeling stage.

In the present study, five plasma metabolites were employed as biomarkers closely tracking the severity of WB. Anserine, a functional dipeptide containing methylhistidine and beta-alanine, is normally present in the brain and skeletal muscles of birds and mammals (71). Anserine increased with the severity of WB. Pectoralis muscle dystrophy was associated with a significantly lower content of anserine in birds (72). The metabolite 1-methylhistidine can potentially serve as a marker for muscle protein turnover and reflect skeletal muscle degeneration and oxidative stress (73, 74). Choline, an essential nutrient for skeletal muscle, is a precursor of Ach, and ion replacement of K^+^ with choline^+^ results in potent inhibition of sarcoplasmic/endoplasmic reticulum Ca^2+^ ATPase in the sarcoplasmic/endoplasmic reticulum of skeletal muscles (75). In addition, 2-oxoarginine is an intermediate of the urea cycle (76), and this metabolite had a higher content in birds with WB. Considering these results, it is evident that WB myopathy deteriorated the capacity for muscle contraction.

According to the Pearson correlation analysis, bacteria-related metabolites participated in the alternation of meat quality and myofiber traits. The metabolite 3-methyladenine can act as an autophagy inhibitor in type 2 diabetes-induced skeletal muscle atrophy (77). Guanidoacetic acid was significantly related to meat quality traits and myofiber traits. The beneficial effect of guanidoacetic acid in meat quality has previously been described and validated. Guanidoacetic acid isolated from gut microbial sources has been confirmed to alleviate the symptoms of WB (78). Therefore, such a drop in gut microbial-related guanidoacetic acid may explain its deficiency in plasma, resulting in negative outcomes for the myofiber area and diameter.

In conclusion, the present study examined the appearance, incidence, meat quality, histopathologic changes, and myofiber parameters in birds with and without wooden breast myopathy. Accordingly, we confirmed that gut microbiota responded to the wooden breast myopathy by driving dynamic changes in the digesta of the plasma involving glutamine and glutamate metabolism and arginine biosynthesis. Our analysis yielded insights into the role of the gut microbiota in birds with mild and severe WB, thereby revealing several potential biomarkers for the WB myopathy diagnosis and treatment. However, there are a few limitations to the current study. Future work could investigate this point about the effects of gut microbial-related metabolites on meat quality and incidence of WB.

## Data Availability Statement

The datasets presented in this study can be found in online repositories. The names of the repository/repositories and accession number(s) can be found in the article/supplementary material.

## Ethics Statement

All animals procedures in this paper were performed under the guidelines for care and use of laboratory animals of hunan agricultural university and approved by animal ethics committee of hunan agricultural university (GBT2018).

## Author Contributions

KK, ZS, and XH: conceptualization. NZ and WP: methodology. KK: validation, formal analysis, investigation, and writing the original manuscript. KK, FP, NZ, WP, MM, LL, FF, SX, HZ, ZS, and XH: review and editing. ZS and XH: supervision. XH: funding acquisition. All authors contributed to the article and approved the submitted version.

## Funding

This study was supported by the National Natural Science Foundation of China (Grant No: 31872378) and the Scientific and technical talents in Hunan Province (2020TJ-Q02).

## Conflict of Interest

The authors declare that the research was conducted in the absence of any commercial or financial relationships that could be construed as a potential conflict of interest.

## Publisher's Note

All claims expressed in this article are solely those of the authors and do not necessarily represent those of their affiliated organizations, or those of the publisher, the editors and the reviewers. Any product that may be evaluated in this article, or claim that may be made by its manufacturer, is not guaranteed or endorsed by the publisher.
